# Intraoperative protective ventilation in patients undergoing major neurosurgical interventions: a randomized clinical trial

**DOI:** 10.1186/s12871-021-01404-8

**Published:** 2021-06-30

**Authors:** Federico Longhini, Laura Pasin, Claudia Montagnini, Petra Konrad, Andrea Bruni, Eugenio Garofalo, Paolo Murabito, Corrado Pelaia, Valentina Rondi, Fabrizio Dellapiazza, Gianmaria Cammarota, Rosanna Vaschetto, Marcus J. Schultz, Paolo Navalesi

**Affiliations:** 1 Anesthesia and Intensive Care, Department of Medical and Surgical Sciences, “Magna Graecia” University, Catanzaro, Italy; 2 Anesthesia and Intensive Care, University Hospital of Padua, Via Giustiniani 2, Padova, Italy; 3 Anesthesia and Intensive Care, “Maggiore Della Carità” Hospital, Novara, Italy; 4 Department of Clinical and Experimental Medicine, University of Catania, Catania, Italy; 5 Anesthesia and Intensive Care, Sant’Andrea Hospital, ASL VC, Vercelli, Italy; 6 Department of Intensive Care, Academic Medical Center, Amsterdam, The Netherlands; 7 Laboratory of Experimental Intensive Care and Anesthesiology (LEICA), Academic Medical Center, Amsterdam, The Netherlands; 8 Anesthesiology and Intensive Care Unit, Department of Medicine-DIMED, University of Padova, Padova, Italy

**Keywords:** Mechanical ventilation, Postoperative pulmonary complications, Neurosurgery

## Abstract

**Background:**

Post-operative pulmonary complications (PPC) can develop in up to 13% of patients undergoing neurosurgical procedures and may adversely affect clinical outcome. The use of intraoperative lung protective ventilation (LPV) strategies, usually including the use of a low V_t_, low PEEP and low plateau pressure, seem to reduce the risk of PPC and are strongly recommended in almost all surgical procedures. Nonetheless, feasibility of LPV strategies in neurosurgical patients are still debated because the use of low Vt during LPV might result in hypercapnia with detrimental effects on cerebrovascular physiology. Aim of our study was to determine whether LPV strategies would be feasible compared with a control group in adult patients undergoing cranial or spinal surgery.

**Methods:**

This single-centre, pilot randomized clinical trial was conducted at the University Hospital “Maggiore della Carità” (Novara, Italy). Adult patients undergoing major cerebral or spinal neurosurgical interventions with risk index for pulmonary post-operative complications > 2 and not expected to need post-operative intensive care unit (ICU) admission were considered eligible. Patients were randomly assigned to either LPV (Vt = 6 ml/kg of ideal body weight (IBW), respiratory rate initially set at 16 breaths/min, PEEP at 5 cmH2O and application of a recruitment manoeuvre (RM) immediately after intubation and at every disconnection from the ventilator) or control treatment (Vt = 10 ml/kg of IBW, respiratory rate initially set at 6–8 breaths/min, no PEEP and no RM). Primary outcomes of the study were intraoperative adverse events, the level of cerebral tension at dura opening and the intraoperative control of PaCO_2._ Secondary outcomes were the rate of pulmonary and extrapulmonary complications, the number of unplanned ICU admissions, ICU and hospital lengths of stay and mortality.

**Results:**

A total of 60 patients, 30 for each group, were randomized. During brain surgery, the number of episodes of intraoperative hypercapnia and grade of cerebral tension were similar between patients randomized to receive control or LPV strategies. No difference in the rate of intraoperative adverse events was found between groups. The rate of postoperative pulmonary and extrapulmonary complications and major clinical outcomes were similar between groups.

**Conclusions:**

LPV strategies in patients undergoing major neurosurgical intervention are feasible. Larger clinical trials are needed to assess their role in postoperative clinical outcome improvements.

**Trial registration:**

registered on the Australian New Zealand Clinical Trial Registry (www.anzctr.org.au), registration number ACTRN12615000707561.

**Supplementary Information:**

The online version contains supplementary material available at 10.1186/s12871-021-01404-8.

## Introduction

About 230 millions patients worldwide undergo major surgical procedures every year, requiring general anaesthesia and invasive mechanical ventilation [1]. Post-operative pulmonary complications (PPC), including atelectasis, pneumonia or infections, can develop in up to 13% of patients undergoing neurosurgical procedures and they may adversely affect the clinical outcome [2, 3].

Different strategies of mechanical ventilation with high tidal volumes (Vt > 10 mL/Kg of predicted body weight), with exposition to elevated airway pressures and without positive end-expiratory pressure (PEEP), are associated with the development of PPC [4]. On the contrary, intraoperative lung protective ventilation (LPV) strategies, usually including the use of a low V_t_, low PEEP and low plateau pressure, seem to reduce the risk of PPC and are strongly recommended [5]. Noteworthy, the PERISCOPE study showed that the development of PPCs was associated with worse outcome [6]. Furthermore, different studies showed that even mild PPCs resulted in increased postoperative mortality, need for intensive care unit (ICU) admission, and ICU and hospital length of stay [2, 7]. Therefore, the use of LPV strategies is becoming more and more frequently adopted in all surgical procedures. Nonetheless, neurosurgical patients were often excluded from most trials on protective intraoperative ventilation because the use of low Vt during LPV might result in hypercapnia with detrimental effects on cerebrovascular physiology [8, 9].

Scientific evidence is still inconclusive on the feasibility of the use of LPV strategies in cranial and spinal surgery [10, 11]. Therefore, the present pilot, assessor-blinded randomized clinical trial was conducted to determine whether a strategy of LPV would be feasible in adult patients undergoing cranial or spinal surgery.

## Materials and methods

### Design and oversight

The study was a single-centre, pilot randomized clinical trial conducted from December 2014 to December 2015 at the University Hospital “Maggiore della Carità” (Novara, Italy). The study was approved by the local Ethics Committee (protocol n° 134/14) and registered at ACTRN (ACTRN12615000707561). Written informed consent was obtained from each participant or from a legal representative, according to the local regulations and to the principles of Helsinki Declaration. The CONSORT recommendations concerning the report of randomized trials were followed [12].

### Patients

Patients were considered to be eligible for participation if they satisfied the following criteria: 1) age > 18 years; 2) undergoing cerebral or spinal neurosurgical interventions with an expected duration > 4 h; 3) risk index for pulmonary post-operative complications > 2 [13]; 4) not expected to need post-operative ICU admission. The preoperative risk index for pulmonary complications is a validated tool to identify patients at risk for postoperative pneumonia [13]. The risk index stratifies patients in risk classes ranging from 1 to 5, with higher risk classes indicating a higher risk of postoperative pulmonary complications [13]. By adding points from single risk factors, the investigators obtained a score associated with a risk class. The single points for risk factors and classification for classes are listed in the Table E1 (in the ESM).

Patients were planned for post-operative ICU admission after a joint team decision, weighing the preoperative clinical conditions (i.e. presence of comorbidities, functional level and issues related to the type and site of lesion), the risk for perioperative complications and postoperative requirements (neuromonitoring, sedation, two-stage surgical intervention).

Exclusion criteria were: 1) history of mechanical ventilation in the previous 2 weeks [14]; 2) Body Mass Index ≥35 kg/m^2^; 3) history of sepsis or acute respiratory failure in the previous 2 weeks [14]; 4) need for emergency surgery; 5) presence of neuromuscular diseases; 6) refusal to participate.

### Randomization and interventions

A computer-generated randomization list was created by an independent investigator. Randomization was conducted using sealed, sequentially numbered, and opaque envelopes placed in the nurse-head office of the operating room and without any stratification factor. Patients who satisfied all inclusion criteria and had no exclusion criteria were randomly assigned in a 1:1 ratio to either LPV or control treatment with a permuted-block method. The two tested ventilation strategies were:

1. Interventional group: low tidal volume with PEEP (LPV strategy) with Vt equal to 6 ml/kg of ideal body weight (IBW), respiratory rate initially set at 16 breaths/min, PEEP at 5 cmH_2_O and application of a recruitment manoeuvre (RM) immediately after intubation and at every disconnection from the ventilator (interventional group).
2. Control group: Vt equal to 10 ml/kg of IBW, respiratory rate initially set at 6–8 breaths/min, no PEEP and no RM. The initial respiratory rate was left to the free decision of the attending anaesthesiologist.

In both groups, the initial inspired oxygen fraction (FiO_2_) set at the ventilator was 0.3. IBW was computed according to the following formula: 50 + 0.91 x (centimeters of height – 152.4) for males and 45.5 + 0.91 x (centimeters of height – 152.4) for females [14].

For safety reasons, in both groups respiratory rate was modulated to achieve physiological pH and normocapnia, as assessed either by Arterial Blood Gases (ABG) (aimed between 35 and 45 mmHg) or through the end-tidal carbon dioxide (etCO_2_) (aimed to range between 30 and 40 mmHg). A transitory increase of the FiO_2_ up to 100% was allowed for safety reasons in case of peripheral oxygen saturation (SpO_2_) < 90%. In case of persistent hypoxemia, as defined by a ratio between arterial partial pressure of oxygen (PaO_2_) and FiO_2_ (PaO_2_/FiO_2_) < 250 mmHg, a RM was applied in both groups. RM was delivered by applying in Pressure Support Ventilation (PSV) mode a PEEP of 30 cmH_2_O for 30 s, without any inspiratory support.

All patients were ventilated with a Flow-I ventilator (Maquet Critical Care, Solna, Sweden).

Intra-operative anaesthesiologic managements were standardized in both groups (see ESM for further details).

### Outcomes

Primary outcomes of the study; intraoperative adverse events, such as i) haemoglobin desaturation (SpO_2_ < 90% with a FiO_2_ ≥ 40%) ii) hypoxemic events (as detected by ABG and defined by a PaO_2_/FiO_2_ < 250 mmHg), and iii) hemodynamic events characterized by hypotension (i.e.*,* mean arterial pressure < 50 mmHg or a remarkable reduction in systolic arterial blood pressure less than 90 mmHg) [14–16], hypertension (i.e.*,* mean arterial blood pressure > 90 mmHg) [16], bradycardia (i.e.*,* heart rate < 50 beats/min) or tachycardia (i.e.*,* heart rate > 95 beats/min); 2) the level of cerebral tension at dura opening; 3) intraoperative control of PaCO_2_ (i.e., number of patients with one or more episodes of hypercapnia and the overall PaCO_2_ detected by ABG).

Intraoperative adverse events were expressed either compositely and separately.

Secondary outcomes were the rate of pulmonary and extrapulmonary complications, the number of unplanned ICU admissions, ICU and hospital lengths of stay and mortality.

Postoperative pulmonary complications were scored using a grade scale ranging from 0 to 4, with grade 0 representing the absence of any pulmonary complication and grades 1 through 4 representing successively the worse forms of complications (see Table E2 in the ESM) [17].

### Data acquisition and analysis

Data were collected through a dedicated case report form and, afterward, uploaded on a customized database on Microsoft Excel (Microsoft Corporation, USA). Patients’ characteristics, administered fluid and blood components, and diuresis were recorded. At the beginning of surgery, ventilator settings, peak (P_peak_) and plateau (P_plat_) pressures were also recorded.

Cerebral tension was scored using a grade scale ranging from 1 to 4, with “1” representing the relaxed brain; “2” representing a mild, acceptable brain swelling; “3” a moderate brain swelling not requiring therapy; “4” indicating a severe swellingrequiring treatment [16]. The level of cerebral tension, as indicated by the surgeon at dural opening was recorded.

The surgeon was blinded with regard to the applied ventilation strategy. The need for osmotic therapies or transient hyperventilation aimed to reduce cerebral tension were also recorded [16]. ABGs were analysed after intubation and at every 1-h interval.

Any adverse event requiring additional monitoring or treatment after patient’s awakening in the recovery room (such as cough, seizure, shivering, haemoglobin desaturation, agitation or altered mental status, uncontrolled severe post-operative pain) and/or during hospital stay, need for ICU admission, hospital length of stay, and mortality were recorded [16]. Moreover, blood test results at baseline, and 1 and 3 days after surgery were collected [14]. Patients were followed by a blinded assessor up to hospital discharge.

### Statistical analysis

For this feasibility study, we chose a small sample size of 60 patients after exclusion and inclusion criteria were fulfilled. However, this sample size allowed us to detect a 32% difference with an alpha error of 10% and a power of 80% in a two-sided test.

All the analyses were performed on an intention-to-treat basis. Normal distribution of data was assessed through the Kolmogorov Smirnov test. Data were expressed as mean (SD) or median [25th–75th IQR], as appropriate. Comparisons of normally distributed variables were performed by unpaired *t-*tests, whereas the Mann-Whitney U-test was used for non-parametric data. Comparisons of two or more proportions were made by using the chi-square test; the Fisher exact test was used for small frequencies. Comparison of ordinal data of cerebral tensions was performed through the Kruskal-Wallis H-test. Multiple data comparison was performed with the analysis of variance (ANOVA) for repeated measures, and the Bonferroni post-hoc test was used, when indicated.

Two-tailed *p* values < 0.05 were considered statistically significant.

## Results

### Patients

**Fig. 1:**
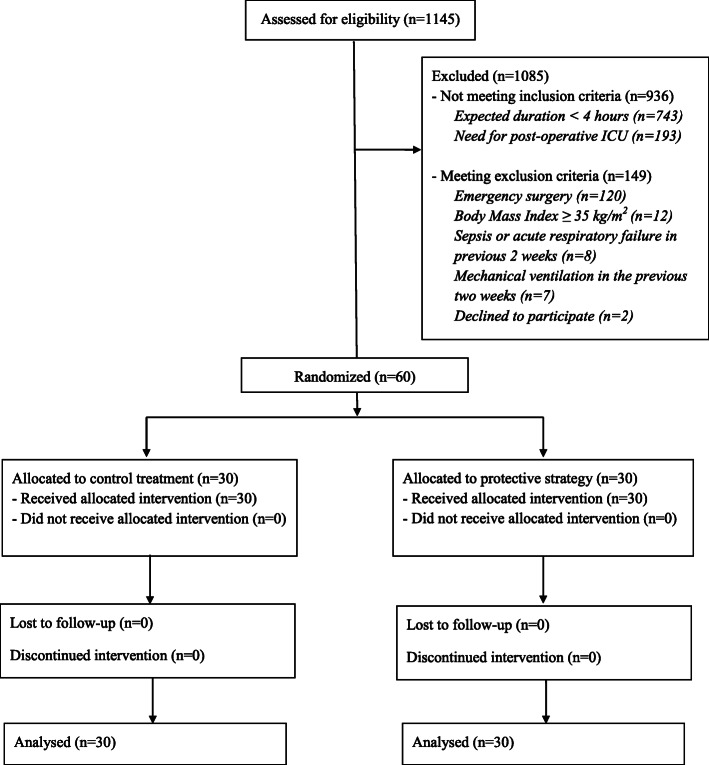
Flowchart of the study. The figure depicts the study flowchart, which includes four arms

From December 2014 through December 2015, a total of 1145 patients scheduled for neurosurgery were assessed for trial eligibility. A total of 60 patients, 30 for each group, were included in the intention-to-treat analysis. All patients received the allocated treatment, and primary outcomes data were available for all patients (Fig. 1).

Baseline characteristics and scheduled surgical procedures were similar between groups (Table 1).

**Table 1:** baseline characteristics of study population

	Control treatment (***n*** = 30)	LPV strategy (***n*** = 30)	***P*** value
Age (years), mean (SD)	58 (13)	58 (18)	0.987
Male, n (%)	15 (50)	12 (40)	0.604
Height (cm), mean (SD)	167 (8)	166 (9)	0.880
Weight (kg), mean (SD)	69.7 (13.0)	69.6 (14.3)	0.978
BMI (kg*m^2^), mean (SD)	25.0 (4.0)	25.1 (4.5)	0.942
ASA Classification, n (%)			
*Class 1*	9	6	0.535
*Class 2*	19	22	
*Class 3*	2	2	
*Class 4*	0	0	
Type of surgery, n (%)			
*Brain surgery*	21 (70)	23 (77)	0.771
*Spinal surgery*	9 (30)	7 (23)	

### Perioperative procedures

Table E3 (in ESM) enlists the intraoperative fluid balance and ventilator settings in both groups. Administered fluids, blood components, diuresis and intraoperative fluid balance were similar between cohorts.

Respiratory rate required to achieve physiological pH and normocapnia was significantly lower in control group, compared to LPV strategy (8 ± 1 vs. 16 ± 1 breaths/min, respectively; *p* < 0.0001). P_peak_ and P_plat_ did not differ between groups (Table E3 in the ESM).

Surgery duration (260 ± 74 vs. 250 ± 98 min, *p* = 0.637) and the time spent under invasive mechanical ventilation (373 ± 96 vs. 393 ± 116 min, *p* = 0.482) were also similar between groups.

All patients received intravenous analgesia and achieved a good control of pain, thus not needing the adjunctive administration of analgesics.

No adverse events after patient’s awakening in the recovery room were recorded.

### Outcomes

No difference in the rate of composited and separated intraoperative adverse events was found between groups (Table 2). Of note, desaturation mainly occurred during the first hour of surgery in the control group, whereas transient hypotensive events were observed during execution of RMs and spontaneously recovered at the end of RMs.

**Table 2:** Primary Outcomes

	Control treatment	LPV strategy	***P*** value
***Patients with one or more adverse events,*** **n (%)**			
Composite	8 (26.7)	8 (26.7)	0.999
Hypoxemic events	6 (20.0)	2 (6.7)	0.129
Haemoglobin desaturation	0 (0.0)	0 (0.0)	0.999
Hypotension or bradycardia	2 (6.7)	6 (20.0)	0.129
***Cerebral tension by the surgeon (only in case of brain surgery)***			
Grade 1 n (%)	7 (33.3)	7 (30.4)	0.677
Grade 2 n (%)	9 (42.9)	10 (43.5)	
Grade 3 n (%)	5 (23.8)	6 (26.1)	
Grade 4 n (%)	0 (0.0)	0 (0.0)	
***PaCO***_***2***_ ***control***			
Patients with one or more episode of hypercapnia n (%)	1 (3.3)	2 (6.6)	0.554
Overall PaCO_2_ recorded (mmHg), mean (SD)	35.5 (4.0)	37.1 (3.4)	0.002

During brain surgery, the grade of cerebral tension was similar between patients randomized to receive control treatment or LPV strategies, as well as the need for osmotic therapy (mannitol 18%) (107 ± 18 mL vs 138 ± 19 mL; *p* = 0.224). (Table 2).

**Fig. 2:**
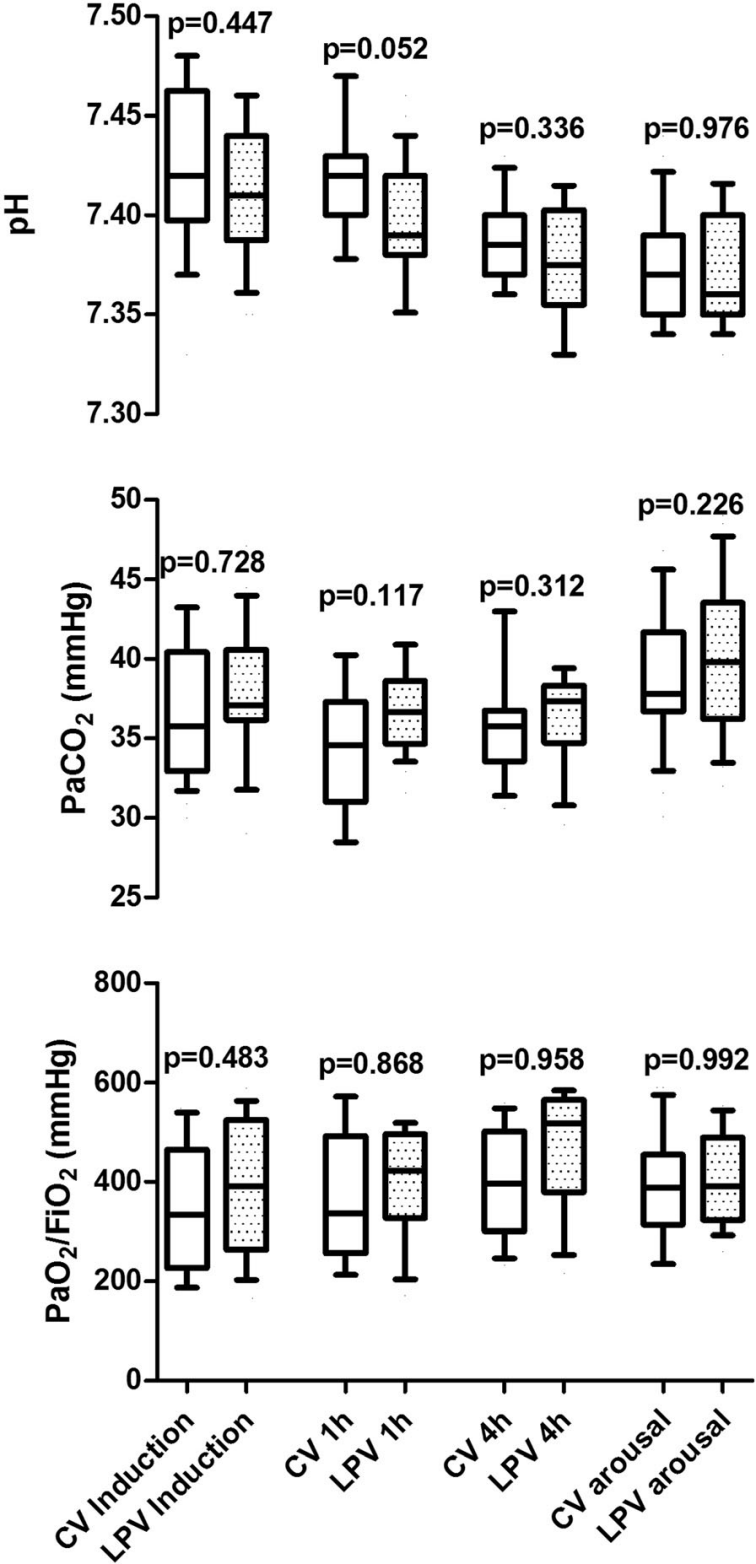
Arterial Blood Gases. Box plots of pH, arterial partial pressure of carob dioxide (PaCO2) and the ratio between arterial partial pressure to inspired fraction of oxygen (PaO2/FiO2) are depicted for control treatment and lung protective (LPV) ventilation strategies groups, at induction, 1 and 4 h after induction and at the extubation. The bottom and top of the box indicate the 25th and 75th percentile, the horizontal band near the middle of the box is the median, and the ends of the whiskers represent the 10th and 90th percentiles. *P* values between study arms are report in the figures

The number of patients who experienced one or more episodes of hypercapnia was also similar between groups. (Table 2) Furthermore, the overall PaCO_2_ was slightly, although significantly, higher in the LPV group, compared to control one (Table 2). ABGs during surgery and at arousal are shown in Fig. 2. Throughout the study protocol, ABGs were similar between the two ventilator strategies.

The rate of postoperative pulmonary and extrapulmonary complications was similar between groups (Table 3). Major clinical outcomes were similar between groups (Table 4).

**Table 3:** Secondary outcomes

	Control treatment (***n*** = 30)	LPV strategy (***n*** = 30)	***P*** value
***Pulmonary complication n (%)***			
Overall	9 (30.0)	7 (23.3)	0.341
*Grade 1*			
Dyspnea not due to other documented cause	0 (0.0)	0 (0.0)	0.999
*Grade 2*			
Atelectasis	3 (10.0)	3 (10.0)	0.999
Chest X-ray worsening	4 (13.3)	2 (6.6)	0.389
Need for oxygen therapy	5 (16.7)	2 (6.6)	0.228
Post-operative hypoxia	8 (26.7)	5 (16.7)	0.347
*Grade 3*			
Pneumonia	1 (3.3)	0 (0.0)	0.313
*Grade 4*			
Need for invasive mechanical ventilation	0 (0.0)	0 (0.0)	0.999
Need for CPAP/NIV	0 (0.0)	0 (0.0)	0.999
***Extra-Pulmonary complications***			
Infective complications, n (%)	2 (6.6)	1 (3.3)	0.554

*CPAP* Continuous Positive Airway Pressure, *NIV* Non-Invasive Ventilation

**Table 4:** Clinical outcomes

	Control treatment (***n*** = 30)	Protective strategy (***n*** = 30)	***P*** value
Unplanned ICU admission, n (%)	2 (6.7)^a^	3 (10.0)^a^	0.999
ICU length of stay (days) mean (SD)	0.3 (0.7)	0.3 (0.5)	0.870
Hospital length of stay (days), mean (SD)	8.1 (3.7)	8.6 (3.6)	0.629
ICU mortality, n (%)	0 (0%)	0 (0%)	0.999
Hospital mortality, n (%)	0 (0%)	0 (0%)	0.999

*ICU* Intensive Care Unit

^a^Noteworthy, 1 patient in the control group and 5 patients in protective strategy were admitted also to ICU after surgery because of shortage of subintensive beds due to the admission of other patients after randomization

## Discussion

The findings of our pilot randomized clinical trial can be summarized as follows: a) use of LPV was feasible in a population of patients scheduled to undergo major neurosurgical procedures, since the cerebral tension assessed by the surgeon were not different between groups, while PaCO_2_ was significantly higher during LPV, though the difference was not clinically relevant; b) the rate of adverse events during surgery was not different between groups; c) no difference in the incidence of PPCs and other relevant outcomes was observed.

In several trials, application of LPV in different types of surgery improved the rates of postoperative complications [14, 15, 18], postoperative arterial oxygenation and pulmonary function tests [15], and also decreased acute lung injury and atelectasis [19], as well as the need for reintubation [20], hemodynamic instability, and renal failure [21]. More recently, the enthusiasm for LPV has been mitigated by some studies reporting no outcome differences between low or high tidal volumes [22] and PEEP [23, 24] during major surgeries. In addition, for fear of hypercapnia, the use of LPV strategies in cranial and spinal surgery is still debated because potentially contraindicated in neurosurgical patients [3].

Some trials reported higher PaCO_2_ values during LPV [15, 25]. Indeed, changes of 1 mmHg in PaCO_2_ levels induce modifications ranging from about 2 to 4% in cerebral blood flow, thus also affecting intracranial pressure [8]. Similar effects were also reported for spinal cord blood flow [9]. In this regard, we detected slightly higher overall PaCO_2_ values (+ 1.6 mmHg) during LPV, while PaCO_2_ was similar at the different timepoints; however, the cerebral tension as evaluated by the surgeon at dural opening, the use of osmotic agents, and/or the need for transient hyperventilation were not different between groups. In fact, a rise of 1.6 mmHg of PaCO_2_ translates into an increase of cerebral blood flow of 2.7 ml every 100 g of tissue per minute, that produces a negligible modification of the intracranial pressure [26].

We observed similar rates of adverse events between groups. Of note, in our population, the vast majority of hemodynamic adverse events (i.e.*,* transient hypotension) occurred during execution of RMs and spontaneously recovered at the end of RMs, consistently with previous trials [14, 27]. This can happen when high pressures are applied to the thoracic system, thus reducing both venous return to the heart and cardiac output, and consequently causing a transient hypotension [28]. The optimization of patients volemia before anaesthesia induction and the use of a stepwise recruitment manoeuvre should be considered in the attempt to reduce the hypotensive events during RM. [29] In fact, the occurrence of intraoperative hypotension, even of short (1–3 min) duration, may be associated with impaired outcomes [30].

Furthermore, one or more desaturations occurred in 20% of patients randomized to control treatment, as compared to 6% in the PLV group. Noteworthy, all episodes occurred within the first hour after anaesthesia induction and resolved with the application of a RM, as per study protocol. This is attributable to the application of a RM immediately after intubation and PEEP in the LPV group.

We also recorded a higher diuresis in the LPV group. Of note, in this group, the administered fluids were slightly, though not significantly, higher. Therefore, the intraoperative fluid balance was similar between groups.

Our trial has some strengths. Notably, the trial is characterized by a low risk for detection bias. First of all, having included the “healthiest” patients and the “easiest” cases, we excluded bias arising from baseline clinical characteristics of patients, reducing important confounding factors. In this way, the clinical effect of ventilatory approach is arguably due to the ventilatory approach itself, not to the baseline patients’ comorbidities or the most complex surgical cases. At the same time, this makes difficult to generalize our results to an average population with co-morbidities, that is the most susceptible population to be affected by the ventilatory setting. Moreover, the main outcomes are characterized by objective assessments, such as the ABGs or pre-defined modifications of vital parameters. Furthermore, secondary outcomes were assessed by assessors blinded to the delivered ventilation strategy. In addition, the trial is characterized by a pragmatic nature of the protocol, while maintaining the routine clinical practice. Moreover, the fluid management was standardized by the trial protocol, overcoming the limitations of previous trials [14].

Before outlining our conclusions, some important limitations deserve to be discussed. First of all, our pilot trial aimed to assess feasibility of LPV, rather than differences in outcome variables. Therefore, no conclusions on postoperative clinically relevant outcomes, such the occurrence of PPCs, can be drawn from our small population. In addition, we acknowledge that the preoperative risk index for pulmonary complications is a validated tool to identify patients at risk for postoperative pneumonia, but it is not a good score to evaluate other postoperative complications such as atelectasis or ARDS. In addition, we included both cranial and spinal surgical interventions which are very different settings and deserve to be analysed separately. Moreover, the method we used to evaluate cerebral tension could raise criticism, due to a poor agreement with the subdural pressure assessed with a small catheter and a pressure transducer system [31]. Nonetheless, our method was used in other studies and previously published [16].

Future trials should consider the results from more recent studies on intraoperative ventilation. For example, PEEP should be set individually, tidal volume should be adjusted on the patients’ lung size and on the predicted body weight in order to assure a P_plat_ < 20 cmH_2_O and a driving pressure < 15 cmH_2_O. In addition, high FiO_2_ should be avoided, if unnecessary [32]. Moreover, since our hypotensive events lasted less than 30 s, further trials with proper design and sample size should investigate if these episodes would impair patients’ outcomes. In addition, the possible impairments due to a slight increase in PaCO_2_ may be probably overpassed by the great benefits derived from the reduction of PPCs occurrence [2, 6, 7]. Nonetheless, this result requires further confirmation in larger trials including those patients excluded from our study. Moreover, we did not record the need and use of hyperosmolar therapy in the post-operative period up to 96 h after surgery. Further trials should also investigate this feature.

## Conclusions

In patients scheduled for major neurosurgery, LPV strategy is feasible. Further studies with an adequate sample size should be properly designed and conducted to assess safety and potential clinical outcome improvements, such as the occurrence of postoperative pulmonary and extrapulmonary complications.

## Supplementary Information

**Additional file 1: Table S1.** Preoperative Risk Index [1]. **Table S2.** Grade scale for postoperative pulmonary complications. **Table S3.** Intraoperative fluid balance and ventilator settings.

## Data Availability

The authors will share all of the individual participant data collected during the trial after de-identification, to researchers who provide a methodologically sound proposal. The full protocol and raw data are available at longhini.federico@gmail.com

## Abbreviations

ABGs: Arterial Blood Gases
etCO_2_: End-tidal Carbon dioxide
FiO_2_: Inspired oxygen fraction
IBW: Ideal Body Weight
ICU: Intensive Care Unit
LPV: Lung Protective Ventilation
PaCO_2_: Arterial tension of carbon dioxide
P_peak_: Peak airway pressures
P_plat_: Plateau airway pressures
PEEP: Positive End-Expiratory Pressure
PPC: Post-operative Pulmonary Complications
PSV: Pressure Support Ventilation
RM: Recruitment Manoeuvre
SpO_2_: Peripheral oxygen saturation
Vt: Tidal volume
