# Supplementary material for: Intraoperative protective ventilation in patients undergoing major neurosurgical interventions: a randomized clinical trial
Source: BMC Anesthesiol. 2021 Jun 30;21:184. doi: 10.1186/s12871-021-01404-8 (PMC8241565; doi:10.1186/s12871-021-01404-8)
Supplement: Supplementary file 1 — Additional file 1: Table S1. Preoperative Risk Index [1]. Table S2. Grade scale for postoperative pulmonary complications. Table S3. Intraoperative fluid balance and ventilator settings. [file 12871_2021_1404_MOESM1_ESM.doc]

**ELECTRONIC SUPPLEMENTAL MATERIAL**

**INTRAOPERATIVE PROTECTIVE VENTILATION IN PATIENTS UNDERGOING MAJOR NEUROSURGICAL INTERVENTIONS: A RANDOMIZED CLINICAL TRIAL.**

Federico Longhini, MD; Laura Pasin, MD; Claudia Montagnini, MD; Petra Konrad, MD; Andrea Bruni, MD; Eugenio Garofalo, MD; Paolo Murabito, MD; Corrado Pelaia, MD; Valentina Rondi, MD; Fabrizio Della Piazza, MD; Gianmaria Cammarota, MD, PhD; Rosanna Vaschetto, MD, PhD; Marcus J. Schultz, MD; Paolo Navalesi, MD, FERS.

**Expanded Methods**

***Preoperative risk index for pulmonary complications***

The preoperative risk index for pulmonary complications is a validated tool to identify patients at risk for postoperative pneumonia [1]. The risk index stratifies patients in risk classes ranging from 1 to 5, with higher risk classes indicating a higher risk of postoperative pulmonary complications [1]. By adding points from single risk factors, the investigators obtained a score associated with a risk class. The single points for risk factors and classification for classes are listed in the Table E1.

***Standard procedure***

The anaesthesiologic procedures were those in our clinical practice. One hour before the induction of anaesthesia, patients received midazolam from 1 to 5 mg e.v. Anaesthesia was induced with propofol 2 mg*kg e.v. bolus (for sedation), cisatracurium 0.1-0.2 mg*kg or rocuronium 0.6-1.2 mg*kg ev bolus (for neuromuscular blocking), and remifentanyl 0.15-0.25mg*kg/min in continuous infusion (for analgesia).

An arterial catheter was placed in a radial artery in order to monitor the blood pressure and to sample blood for gas analysis. A central vein catheter was also introduced in an internal jugular vein with the ultrasound-guided technique. During the surgery, sedation was maintained with or sevofluorane with a Minimal Alveolar Concentration (MAC) ranging between 0.75-1.25 [2], or propofol delivered with a dedicated pump for Target Controlled Infusion (TCI) adjusted to obtain a deep sedation as assessed through Bispectral Index (BIS) around 40. Intraoperative analgesia was assured with continuous infusion of remifentanil between 0.05 and 0.25 mg*kg/min [2]. Neuromuscular blockade was assured through continuous infusion of cisatracurium or rocuronium in order to have a complete neuromuscular block as assessed through train-of-four (TOF) monitoring.

At the end of the surgery, patients were awakened after resolution of the neuromuscular blockade (i.e. a TOF ratio ≥0.9). Post-operative analgesia was assured through the administration of 0.03-0.1 mg*kg e.v. of morphine and paracetamol 10-15 mg*kg 90-minutes before the end of surgery. In the first 48 hours after surgery, analgesia was assured with morphine and paracetamol and adjusted in order to have a good control of the pain. Pain was evaluated through an 11-point numeric rating scale (NRS). Briefly, patients were asked to indicate a number between 0 (no pain) and 10 (worst pain) on an adapted, large printed scale including numbers and descriptors. Scores ≥7 were indicating severe pain [2].

Intra-operative fluid management was also standardized. Sixty minutes before surgery, 0.25-0.50 g/kg or 0.70-1.00 g/kg of mannitol were administered if a shift of the median cerebral line was absent or present, respectively [3]. Intra-operative cristalloids (normal saline and acetate ringer) were infused in order to balance the pre-operative fastening, the urinary output and the intra-operative loss of fluids. In particular, the pre-operative fastening was computed as 60ml + 1ml/Kg for every Kg above 20, during the first hours of surgery, while the intra-operative loss of fluids was computed as 3ml*kg/hour of surgery plus blood loss [4]. Blood transfusions, fresh-frozen plasma and platelet concentrates were administered according to the recommendations for the transfusion management of patients in the intra-operative period, released by the Italian Society of Transfusion Medicine and Immunohaematology [5].

***Post-operative complications***

We further recorded the post-operative complications during the hospital stay [6]. In particular, we recorded and scored postoperative pulmonary complications on a grade scale from 0 to 4, as previously described (see Table E2) [6]. Moreover, we recorded:

1) postoperative hypoxemia as defined by a PaO_2_ < 60 mmHg or SpO_2_ < 90% on room air [6];

2) occurrence of pneumonia**,** as suspected upon the presence of new and/or progressive pulmonary infiltrates on chest x-ray plus two or more of the following criteria: a) temperature ≥ 38.5°C or **<**36°C; b) leukocytosis (*i.e.;* ≥ 12000 White Blood Cell (WBC)/mm^3^ or leukopenia <4000 WBC/mm^3^; c) presence of purulent sputum and/or new onset or worsening of cough or dyspnea [6];

3) presence of lung atelectasis, defined by lung opacification with mediastinum, hilum or hemidiaphragm displacement towards the affected area and compensatory over-inflation in the adjacent non-atelectatic lung [6];

4) occurrence of infective complications (i.e.; sepsis, severe sepsis or septic shock), as *a posteriori* defined according to the more recent international consensus [7].

***Randomization***

For this feasibility study, we chose a small sample size of 60 patients after exclusion and inclusion criteria were fulfilled. However, this sample size allowed us to detect a 32% difference with an alpha error of 10% and a power of 80% in a two-sided test.

Patients were randomized to control ventilation or LPV according to a computer-generated sequence in sealed, opaque numbered envelops. The envelopes were kept in the head of nurses' office. Envelopes were opened by the nurse charged of the operating room at the time every patient met all inclusion and not exclusion criteria; the group of assignment was, therefore, communicated to the anaesthesiologists.

*Table E1 Preoperative Risk Index [1]*

| **Risk Factors** | **Points** |
| --- | --- |
| *Type of surgery* |  |
| Abdominal aortic aneurysm repair | 15 |
| Thoracic surgery | 14 |
| Upper abdominal surgery | 10 |
| Neck surgery | 8 |
| Neurosurgery | 8 |
| Vascular surgery | 3 |
| *Age* |  |
| ≥ 80 years/old | 17 |
| 70-79 years/old | 13 |
| 60-69 years/old | 9 |
| 50-59 years/old | 4 |
| *Functional status* |  |
| Totally dependent | 10 |
| Partially dependent | 6 |
| *Weight loss > 10% in the last 6 months* | 7 |
| *Chronic Obstructive Pulmonary Disease* | 5 |
| *General anesthesia* | 4 |
| *Impaired sensorium* | 4 |
| *History of cerebrovascular accident* | 4 |
| *Blood urea nitrogen level* |  |
| <2.86 mmol/L (<8mg/dL) | 4 |
| 7.85-10.7 mmol/L (22-30 mg/dL) | 2 |
| ≥10.7 mmol/L (≥30 mg/dL) | 3 |
| *Transfusion ≥ 4 units* | 3 |
| Emergency surgery | 3 |
| Chronic steroid therapy | 3 |
| Current smoker within 1 year | 3 |
| Alcohol intake > 2 drinks/day in the last two weeks | 2 |

|  | **Risk Class** | | | | |
| --- | --- | --- | --- | --- | --- |
|  | **1** | **2** | **3** | **4** | **5** |
| *Total points* | 0-15 | 16-25 | 26-40 | 41-55 | > 55 |
| *Risk of post-operative pneumonia (%)* | 0.24 | 1.19 | 4.0 | 9.4 | 15.8 |

*Table E2* *Grade scale for postoperative pulmonary complications*

| *Grade 0* | Absence of any pulmonary complication |
| --- | --- |
| *Grade 1* | - Dry cough  - Microatelectasis (*i.e.;* abnormal lung findings and temperature **>** 37.5°C  without other documented cause; results of chest radiograph either normal)  - Dyspnea not due to other documented cause |
| *Grade 2* | - Productive cough not due to other documented cause  - Bronchospasm (*i.e.;* new wheezing or pre-existent wheezing resulting in change therapy)  - Hypoxemia (*i.e.;* PaO_2_ <60mmHg or SpO_2_ <90% in room air)  - Atelectasis (*i.e.;* radiological confirmation plus either temperature **>** 37.5°C or  abnormal lung findings)  - Transient hypercarnia requiring treatment |
| *Grade 3* | - Pleural effusion resulting in thoracentesis  - Suspected (*i.e.;* radiological evidence without bacteriological confirmation) or proved pneumonia (*i.e.;* radiological evidence and documentation of pathological organism by Gram stain or culture)  - Pneumothorax  - Post-operative respiratory failure requiring non-invasive or invasive ventilation for less than 48 hours |
| *Grade 4* | - Post-operative respiratory failure requiring non-invasive or invasive ventilation for at least 48 hours |

*Table E3: Intraoperative fluid balance and ventilator settings*

|  | **Controls (n=30)** | **LPV strategy (n=30)** | **P value** |
| --- | --- | --- | --- |
| *Fluid Balance* |  |  |  |
| Fluid administrated (ml) | 3450 [2500; 4300] | 3950 [3075; 4563] | 0.217 |
| Cristalloids | 3450 [2500; 4300] | 3950 [3075; 4563] | 0.217 |
| Colloids | 0 [0; 0] | 0 [0; 0] | 0.999 |
| Blood transfusion (ml) | 0 [0; 0] | 0 [0; 0] | 0.544 |
| Diuresis (ml) | 1250 [995; 2000] | 1920 [1438; 2413] | 0.032 |
| Overall fluid balance (ml) | 255 [-29; 365] | 164 [90; 302] | 0.595 |
|  |  |  |  |
| *Ventilator settings* |  |  |  |
| Tidal volume (ml) | 599 (98) | 357 (51) | <0.0001 |
| Respiratory rate (breaths/min) | 8 (1) | 16 (1) | <0.0001 |
| P_peak_ (cmH_2_O) | 18.6 (2.3) | 18.8 (3.2) | 0.779 |
| P_plat_ (cmH_2_O) | 14.3 (2.4) | 15.1 (3.0) | 0.228 |
| PEEP (cmH_2_O) | 0.0 (0.0) | 5.0 (0.0) | <0.0001 |
| Driving Pressure (cmH_2_O) | 14.3 (2.4) | 10.1 (3.0) | <0.0001 |
| Recruitments manoeuvres (n for patient) | 0.4 (0.8) | 2.1 (0.3) | <0.0001 |

P_peak_, inspiratory peak airway pressure; P_plat_, plateau airway pressure; PEEP, Positive End-Expiratory Pressure. Data are expressed as mean (standard deviation) or median [25^th^; 75^th^ range].

**References**

1. Arozullah AM, Khuri SF, Henderson WG, Daley J: **Development and validation of a multifactorial risk index for predicting postoperative pneumonia after major noncardiac surgery**. *Ann Intern Med* 2001, **135**(10):847-857.

2. Citerio G, Pesenti A, Latini R, Masson S, Barlera S, Gaspari F, Franzosi MG: **A multicentre, randomised, open-label, controlled trial evaluating equivalence of inhalational and intravenous anaesthesia during elective craniotomy**. *Eur J Anaesthesiol* 2012, **29**(8):371-379.

3. Quentin C, Charbonneau S, Moumdjian R, Lallo A, Bouthilier A, Fournier-Gosselin MP, Bojanowski M, Ruel M, Sylvestre MP, Girard F: **A comparison of two doses of mannitol on brain relaxation during supratentorial brain tumor craniotomy: a randomized trial**. *Anesth Analg* 2013, **116**(4):862-868.

4. Tommasino C: **Fluids and the neurosurgical patient**. *Anesthesiol Clin North America* 2002, **20**(2):329-346, vi.

5. Liumbruno GM, Bennardello F, Lattanzio A, Piccoli P, Rossetti G: **Recommendations for the transfusion management of patients in the peri-operative period. II. The intra-operative period**. *Blood Transfus* 2011, **9**(2):189-217.

6. Futier E, Constantin JM, Paugam-Burtz C, Pascal J, Eurin M, Neuschwander A, Marret E, Beaussier M, Gutton C, Lefrant JY *et al*: **A trial of intraoperative low-tidal-volume ventilation in abdominal surgery**. *N Engl J Med* 2013, **369**(5):428-437.

7. Singer M, Deutschman CS, Seymour CW, Shankar-Hari M, Annane D, Bauer M, Bellomo R, Bernard GR, Chiche JD, Coopersmith CM *et al*: **The Third International Consensus Definitions for Sepsis and Septic Shock (Sepsis-3)**. *JAMA* 2016, **315**(8):801-810.
